# Intratumoral CD73: An immune checkpoint shaping an inhibitory tumor microenvironment and implicating poor prognosis in Chinese melanoma cohorts

**DOI:** 10.3389/fimmu.2022.954039

**Published:** 2022-09-05

**Authors:** Zixu Gao, Lu Wang, Zhengqing Song, Ming Ren, Yang Yang, Jianrui Li, Kangjie Shen, Yinlam Li, Yiteng Ding, Yanwen Yang, Yuhong Zhou, Chuanyuan Wei, Jianying Gu

**Affiliations:** ^1^ Department of Plastic Surgery, Zhongshan Hospital, Fudan University, Shanghai, China; ^2^ Department of Medical Oncology, Zhongshan Hospital, Fudan University, Shanghai, China

**Keywords:** melanoma, CD73, PD-L1, immunotherapy, CD8^+^ T cells, immunosuppressive, prognosis, tumor microenvironment

## Abstract

**Background:**

As a novel immune checkpoint, CD73 has been reported to play prominent roles in several malignancies. However, the significance of CD73 in melanoma remains ambiguous. This study sought to reveal the impact of CD73 on the tumor microenvironment (TME) and patients’ prognosis, and to investigate whether CD73 could be a therapeutic target in Chinese melanomas, which were dominated by acral and mucosal subtypes.

**Methods:**

Two independent Chinese cohorts of 194 patients with melanoma were enrolled. CD73 and PD-L1 expression as well as CD8^+^ and CD56^+^ cell infiltrations were evaluated by immunohistochemistry in 194 resected melanoma samples. Clinical outcomes of patients were assessed utilizing the Kaplan-Meier plotter and Cox proportional hazard analysis. RNA-seq data was obtained from TCGA database. Gene set functional annotations were performed based on GO, KEGG and GSEA analysis. CIBERSORT, ssGSEA and TIMER were used to explore the association between CD73 and immune infiltration. These findings were validated by establishing tumor xenograft model, and functions of tumor-infiltrating immune cells were examined by flow cytometry and immunofluorescence.

**Results:**

High CD73 expression showed poorer clinical outcomes and was identified as an independent prognostic indicator for survival in two cohorts. Expression of CD73 was more prevalent than PD-L1 in Chinese melanoma cohorts (54.6% vs 23.2%). Co-expression of both immune checkpoints was infrequent (12.9%) in melanoma, and 54.4% of PD-L1 negative cases showed elevated expression of CD73. CD73^high^ tumors showed a microenvironment with fewer CD8^+^ T cells and CD56^+^ NK cells infiltration, which displayed a dysfunctional phenotype. With the treatment of CD73 inhibitor APCP, the amount of CD8^+^ T cells and CD56^+^ NK cells infiltrated in tumors was elevated and the immunosuppressive effect of CD73 was eliminated.

**Conclusions:**

High CD73 expression was associated with an inhibitory TME and adverse clinical outcomes of melanoma. In comparison to PD-L1, CD73 was more prevalent and possessed more definite prognostic significance. Therefore, it may serve as a prognostic indicator and immunotherapeutic target next to PD-L1 in melanoma for Chinese population.

## Introduction

Melanoma is the most lethal type of cutaneous malignancy worldwide (1), accounting for approximately 324,635 new cases and 57,043 deaths in 2020 (2). The incidence of melanoma has been on the rise in recent years, at a rate of 3-5% annually (3). Surgical resection is effective for early-stage melanoma, but most patients are diagnosed at advanced stages, with a median survival of 6-9 months and 5-year overall survival (OS) rate of 30%~40% (4). Despite the unprecedented clinical benefits brought by tumor immunotherapy (5), their prolongation of survival remains unsatisfactory (6, 7). Consequently, it is urgently required to develop novel therapeutic targets that can significantly benefit patients with melanoma.

Over the past few years, immunotherapy has revolutionized the treatment of solid tumors, achieving dramatic improvement in patient survival (8–11). There is growing evidence that the tumor microenvironment (TME) is essential for tumorigenesis, tumor progression and regulation of immune responses (12). Immunotherapy that restore defective immune responses in the TME, such as immune checkpoint inhibitors, have been proved to induce sustained antitumor response in several relapsed and refractory malignancies (13, 14). As a touchstone, melanoma therapeutics have been at the cutting edge of immuno-oncology and immunotherapy. Thus, it is essential to investigate the TME of melanoma. Currently, anti-PD-1/PD-L1 therapy has been approved by FDA as adjuvant therapy for resected melanoma, and tumor PD-L1 expression was proposed as a potential biomarker for predicting response to anti-PD-1/PD-L1 therapy (13). However, given the high heterogeneity of melanoma, the proportion of patients who respond to anti-PD-1/PD-L1 therapy remains modest, and some patients even exhibit accelerated tumor progression after therapy (15).

Acral and mucosal melanoma are the major subtypes of melanoma in China, rather than cutaneous melanoma, which is predominant in the West (16). Thus, there are significant differences of molecular, genomic and immunologic characteristics between Asian and Caucasian populations, which may induce the lower efficacy of PD-1 inhibitor in the Chinese compared to Western population (17–19). The clinical efficacy of PD-1 blockade cannot be generalized to melanoma globally. Therefore, other immune checkpoints are required to be investigated to provide more options for melanoma patients excluded from anti-PD-1/PD-L1 therapy.

CD73, which is also known as ecto-5’-nucleotidase (*NT5E*), is a cell surface enzyme. CD73 works in tandem with its upstream molecule CD39 to catalyze the hydrolysis of extracellular AMP into extracellular adenosine (eADO), which subsequently exerts immunosuppressive effects by accumulating and binding to adenosine receptor (20, 21). Meanwhile, as a newly identified immune checkpoint, CD73 is overexpressed in the TME (22). Several studies suggested that CD73 may represent a potential clinical biomarker, and is related to poor prognosis and tumor progression in a variety of tumors (23–25). Additionally, serum CD73 was found to be closely correlated with the clinical efficacy of PD-1 inhibitor in metastatic melanoma (26). Despite the advances in exploring the role CD73 played in tumor development and progression, the expression pattern, clinical value and the impact of CD73 on the TME remain obscure in melanoma. These have garnered significant attention and indicate that CD73 may represent a promising target next to PD-L1 for tumor immunotherapy.

Given the role of CD73, in the present study we aimed to clarify the impact of CD73 on prognosis, TME, tumor immune infiltration and immunotherapy in melanoma. Our findings would contribute to disclosing the multi-faceted roles of CD73 and the potential association between CD73 and inhibitory TME, which might have implications for future immunotherapy in melanoma.

## Materials and methods

### Patient selection and follow-up procedures

194 patients underwent total resection for melanoma in the Department of Plastic & Reconstructive Surgery of Zhongshan Hospital, Fudan University (Shanghai, China) were randomly allocated into discovery and validation sets. Patients were included if they met the following criteria: (1) histologically and pathologically confirmed melanoma; (2) received no form of radiotherapy or chemotherapy before the operation; (3) no history and concurrence of other malignancies; (4) complete resection of tumors with microscopically negative resection margins; (5) complete clinicopathological and follow-up data. This study was approved by the Ethics Committee of the Zhongshan Hospital Biomedical Research Department. The discovery and validation sets comprise 90 and 104 patients, respectively. The baseline characteristics of the discovery and validation sets are detailed in **Table 1**.

**Table 1:** Correlations between CD73 and baseline clinicopathologic features in melanoma patients.

Variable	Discovery set (n=90)					Validation set (n=104)				
	Patients		CD73 expression			Patients		CD73 expression		
	NO.	%	Low	High	*p*-value	NO.	%	Low	High	*p*-value
All patients	90	100.0	40	50		104	100	48	56	
Age					0.981					0.349
<60	37	41.1	17	20		43	41.3	17	26	
≥60	53	58.9	23	30		61	58.7	31	30	
Gender					0.310					0.964
Male	47	52.2	18	29		55	52.9	26	29	
Female	43	47.8	22	21		49	47.1	22	27	
Anatomic site					0.188					0.625
Acra	49	54.4	26	23		58	55.8	29	29	
Trunk	22	24.4	7	15		25	24.0	11	14	
Other	19	21.1	7	12		21	20.2	8	13	
Histologic type					0.274					0.102
Superficial spreading	23	25.6	10	13		31	29.8	18	13	
Nodular	18	20.0	5	13		21	20.2	5	16	
Acral	28	31.1	16	12		34	32.7	17	17	
Lentigo maligna	21	23.3	9	12		18	17.3	8	10	
Breslow depth (mm)					0.013					0.126
≤2	51	56.7	29	22		49	47.1	27	22	
>2	39	43.3	11	28		55	52.9	21	34	
Clark level					0.036					0.047
I– III	44	48.9	25	19		53	51.0	30	23	
IV – V	46	51.1	15	31		51	49.0	18	33	
Ulceration					0.676					0.766
Present	21	23.3	8	13		13	12.5	7	6	
Absent	69	76.7	32	37		91	87.5	41	50	
Lymph nodes metastasis					0.265					0.526
No	73	81.1	35	38		84	80.8	37	47	
Yes	17	18.9	5	12		20	19.2	11	9	
Distant metastasis					0.155					0.283
No	69	76.7	34	35		76	73.1	38	38	
Yes	21	23.3	6	15		28	26.9	10	18	
Clinical stage					0.023					0.009
I– II	62	68.9	33	29		56	53.8	33	23	
III – IV	28	31.1	7	21		48	46.2	15	33	

A chi-square test was used for comparing groups between low and high CD73 expression. p < 0.05 was considered significant.

### Tissue microarray (TMA) construction, immunohistochemistry (IHC) and immunofluorescence (IF)

The construction of TMA was performed as the procedure described previously (27). To sum up, surgical specimens were fixed in formalin, embedded in paraffin and then HE stained for selection of representative tumor areas. Duplicate cores of 1mm diameter were representative of tumor from each patient.

IHC assay was performed as described in our previous study (28). Briefly, slides were baked, deparaffinized and rehydrated. After being incubated in 0.3% H_2_O_2_, antigen retrieval was performed. Subsequently, the sections were incubated with the primary antibody at 4°C overnight, horseradish peroxidase-labeled secondary antibody (Gene Tech, Shanghai, China). Then, the sections were stained with diaminobenzidine, counterstained with hematoxylin, dehydrated in ethanol, cleared in xylene, and cover-slipped with resin.

To perform IF, slides were prepared in the same procedure as for IHC before incubation of antibodies. The staining was performed as described previously (29). In brief, the slides were subsequently fixed with 4% paraformaldehyde, incubated in 0.3% Triton X-100 and blocked with 5% FBS. Then, the sections were incubated with primary antibodies at 4°C overnight, followed by incubation with secondary antibody (Yeasen, Shanghai, China). The nuclei were counterstained with 4, 6-diamidino-2-phenylindole (DAPI, Yeasen, Shanghai, China). Details of IHC and IF antibodies were shown in **Supplementary Table 1**.

### Quantification of CD73, PD-L1 and infiltration of CD8^+^ T cells and CD56^+^ NK cells

The images of IHC and IF staining of all slides were obtained using the standard microscope (Olympus, Tokyo, Japan) or the CaseViewer software (3DHISTECH, Budapest, Hungary). For staining quantification, Image-Pro Plus software (V 6.0, Rockville, USA) was used to evaluate CD73, PD-L1, CD8 and CD56 expression in the digital photograph expression, and uniform settings were applied for all slides. Slides were evaluated by two pathologists independently and without reference to the patients’ clinical information. Discrepant results between investigators were resolved by reaching a consensus.

The score for CD73 staining was determined based on a combination of staining percentage and intensity as previously described (30). The staining intensity was scored as follows: 0 (negative), 1 (weak), 2 (moderate), or 3 (strong), and the staining proportion was scored as follows: 1 (0−25%), 2 (>25−50%), 3 (>50−75%) or 4 (>75−100%). The sum was used for evaluating the expression level of CD73 and it was classified into two grades: low (0−3) or high (4−7) expression.

### Functional enrichment analysis

Human RNA-Seq data was downloaded from the TCGA database (https://portal.gdc.cancer.gov/; all 471 melanoma cases). Patients were assigned into CD73^high^ (n = 236) and CD73^low^ (n = 235) groups based on the median expression value of gene *NT5E* according to the RNA sequencing data for further analysis and comparison. Differential expression analysis of the two groups was performed using the DESeq2 differential expression library in the R statistical environment. Selection criteria for differentially expressed genes (DEGs) were as follows: |logFC| > 1 and p < 0.05. GO and KEGG enrichment analysis of DEGs were implemented by the enrichplot R Package. Top GO and KEGG categories were selected according to the p-values. GSEA between the two groups was performed with GSEA 2.1.0 with KEGG and HALLMARK database. P < 0.05 was considered statistically significant.

### Analysis of immune cell patterns in tumor microenvironment

The analytical tool, CIBERSORT, was used to analyze the immune cell proportions of all samples from TCGA database. CIBERSORT was developed by Newman et al. (31) which could quantify 22 types of immune cells in tissues according to normalized gene expression profiles. The standardized processed data set of gene expression was uploaded to the CIBERSORT website (https://cibersortx.stanford.edu/index.php). To improve the accuracy of the algorithm, Monte Carlo sampling was employed to deconvolute each sample to determine an empirical CIBERSORT p-value, and only samples with p < 0.05 were deemed appropriate for analysis.

To quantify 29 immune and tumor-related signatures in each sample, ssGSEA analysis was performed using the Gene Set Variation Analysis (GSVA) package of R software, and the infiltration level of different immune cells, immune-related pathways and the activity of immune-related functions in melanoma expression profiles were determined (32). The ssGSEA applied the genetic characteristics expressed by immune cell populations to individual tumor samples (33).

We also reexamined the immune-associated function of CD73 in public data using the Tumor Immune Estimation Resource (TIMER 2.0) (http://timer.comp-genomics.org), which could characterize the association between gene expression and tumor-infiltrating immune cells. With this tool, we explored the relationship between CD73 expression and immune cell infiltration levels in melanoma, including CD8^+^ T cell, activated NK cell, follicular helper T cell and resting memory CD4^+^ T cell.

### Cell culture and transfection

The mouse melanoma cell line B16-F10 was purchased from the Cell Bank of the Chinese Academy of Sciences (Shanghai, China). B16-F10 cells were cultured in DMEM medium (HyClone, Logan, USA) with 10% fetal bovine serum (Gibco, Waltham, USA), streptomycin sulfate (100 μg/ml), and penicillin (100 IU/ml), and were incubated at 37°C with 5% CO_2_. All lentiviral vectors were purchased from OBiO Technology (Shanghai, China). The pSLenti-SFH-EGFP-P2A-Puro-CMV-Nt5e-WPRE lentiviral vectors ware transfected into B16-F10 cells. The transfected cells were screened with puromycin (2 μg/ml) for 1 week to establish stable cell lines.

### *In vivo* assays

All of the animal experiments were approved by the Animal Experimentation Ethics Committee of Zhongshan Hospital, Fudan University. All animals were handled strictly according to the Principles for the Utilization and Care of Vertebrate Animals and the Guide for the Care and Use of Laboratory Animals.

To establish the subcutaneous xenograft tumor models, CD73-NC or CD73-OE B16-F10 cells were subcutaneously inoculated with 5 × 10^6^ cells/mouse on the right flank of six-week-old C57BL/6 mice. Once the tumor was detected, tumor size was measured every 3 days by a vernier caliper and tumor volume was calculated according to the formula volume (mm^3^) =L*W^2^*0.5, where L and W stood for the largest and smallest diameters, respectively. Animals were euthanized when the tumor volumes reached a maximum of 2000mm^3^.

To investigate the effects of CD73 inhibitor APCP, B16-F10 cells were subcutaneously inoculated with 5 × 10^6^ cells/mouse on the right flank of C57BL/6 mice. On day 10 and day 12 following tumor cell implantation, mice were treated with APCP (400 μg/mouse) or PBS by the peritumoral (p.t.) route. The time point was determined according to previous studies since it induced optimal anti-tumor effects (34). Mice were sacrificed on day 13 following B16-F10 injection, and melanoma tissues were isolated for further flow cytometry and immunofluorescence.

### Flow cytometry

Tumor infiltrating cells were quantitatively determined by flow cytometry. Briefly, fresh tissues were harvested as soon as the tumors were resected. Tissues were minced and digested with collagenase IV (Sigma) and then incubated with RBC lysis buffer (BD Biosciences) to lyse red blood cells. The single-cell suspensions were washed and resuspended in phosphate-buffered saline/0.1% bovine serum albumin and then respectively stained with fluorochrome-labeled membrane markers for 40 min at 4°C. The single-cell suspensions were also treated with Fixation/Permeabilization Solution Kit (BD Biosciences) according to manufacture protocol and intracellular proteins were stained by corresponding fluorochrome-labeled antibodies. FACS data were collected *via* BD FACS Celesta and analyzed *via* Flowjo V.10.0 (Tree Star). Details of FCM antibodies were shown in **Supplementary Table 1**.

### Statistical analysis

Chi-squared test, Fisher’s exact test or Mann-Whitney U test were utilized as appropriate to evaluate the associations between CD73, PD-L1 and other variables including densities of different subtypes of immune cells. Kaplan-Meier method and log-rank test were applied to depict the survival curves of OS for patients with different CD73 and PD-L1 expression levels. Univariate and multivariate analysis were performed based on Cox proportional hazard model. P < 0.05 was considered statistically significant. All statistical analysis were performed *via* the SPSS software version 25.0 (SPSS, Inc., Chicago, USA), R software V.4.0.2 (R Foundation for Statistical Computing, http://www.r-project.org/) and GraphPad Prism 8.0 (GraphPad Software, USA).

## Results

### Expression pattern of CD73 and PD-L1 in melanoma

**Figure 1:**
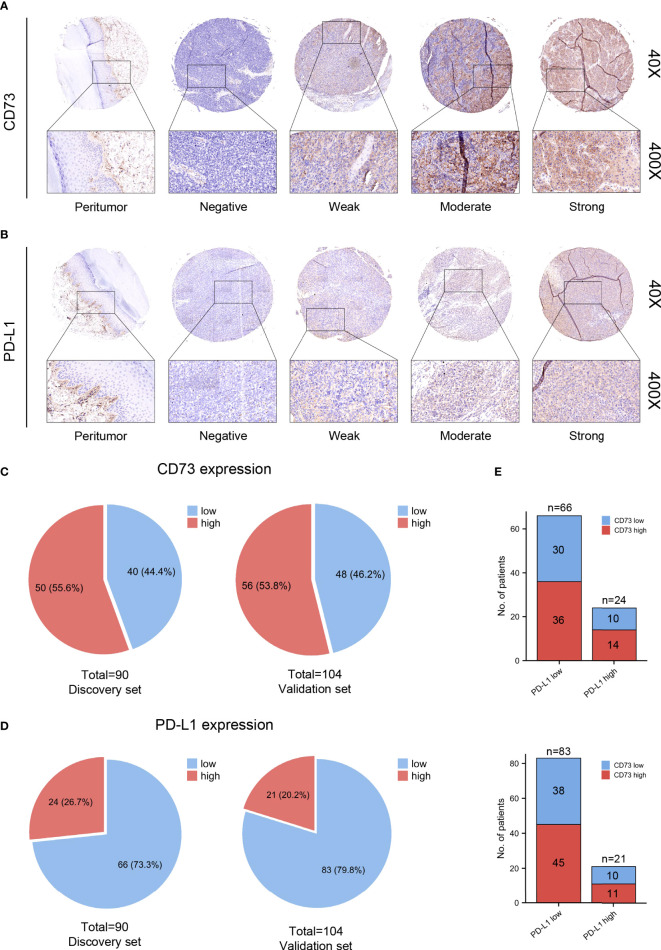
CD73 and PD-L1 expression in melanoma specimens. Representative micrographs of CD73 **(A)** and PD-L1 **(B)** expression within melanoma. The positive rate of CD73 in discovery and validation sets were 55.6 and 53.8% respectively **(C)**. PD-L1 was elevated in 26.7 and 20.2% of cases in discovery and validation sets, respectively **(D)**. No significant correlation was found between CD73 and PD-L1 expression **(E)**.

The correlation between CD73 expression and baseline clinicopathologic features of enrolled patients were described in **Table 1**. We additionally explored the correlation between CD73 expression and the BRAF mutational status utilizing data from GSE158403 and GSE190113 (**Supplementary Figure 1**). As shown in the figure, negative to strong expression of CD73 was observed in melanoma TMAs (**Figure 1A**). The intratumoral tissues showed higher CD73 expression compared to peritumoral tissues, and it was mostly detected on the membrane of tumor cells. Typical micrographs of PD-L1 expression were illustrated in **Figure 1B**. In the discovery set, 55.6% (50/90) and 44.4% (40/90) of the patients were respectively classified as CD73^high^ and CD73^low^ groups. The proportion of patients with high CD73 expression in the validation set was 53.8% (**Figure 1C**). In the discovery and validation sets, the positive rate of PD-L1 were 26.7% (24/90) and 20.2% (21/104) respectively (**Figure 1D**).

No significant associations were identified between CD73 and PD-L1 expression. The co-expression of both immune checkpoints was detected in 12.9% (25/194) cases. In PD-L1 negative melanoma, 54.5% (36/66) and 54.2% (45/83) patients in the discovery and validation sets showed elevated CD73 expression, respectively (**Figure 1E**). In conclusion, CD73 expression was more frequent in melanoma than PD-L1.

### Prognostic significances of CD73 and PD-L1

**Figure 2:**
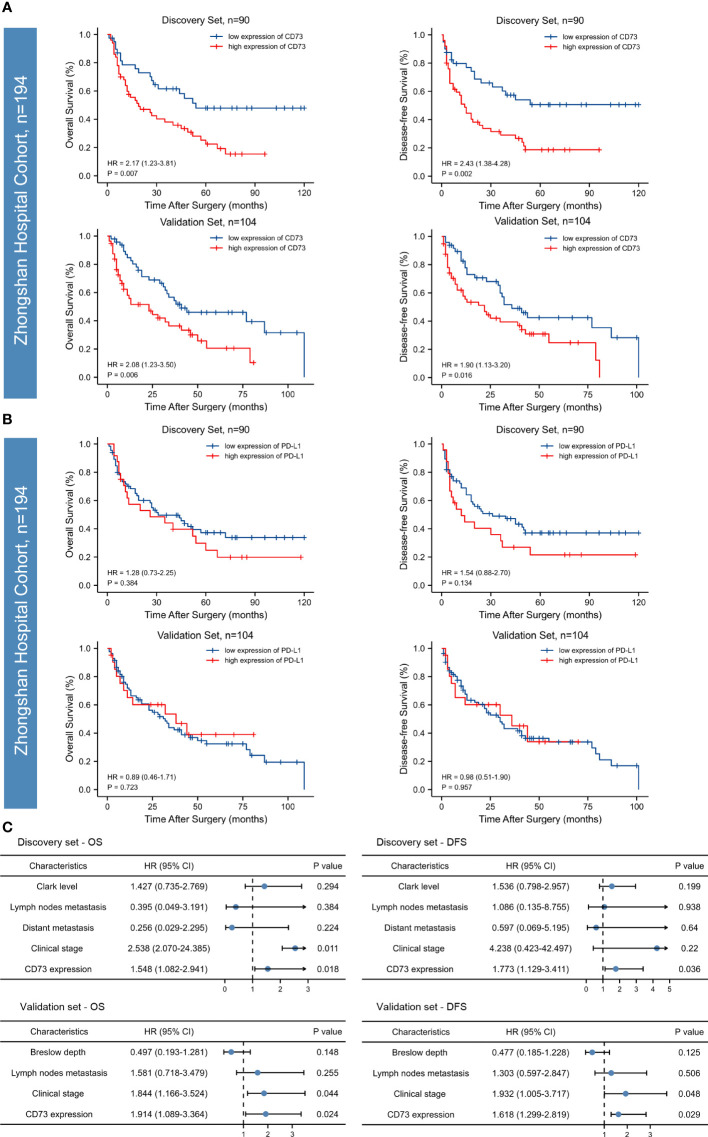
Kaplan-Meier survival curves and forest plots for OS and DFS of patients with melanoma. High CD73 expression was significantly associated with poor overall survival (OS) and disease-free survival (DFS) in the discovery set and the relevance was confirmed in an independent validation set **(A)**. PD-L1 expression in both of the discovery and validation sets failed to stratify OS and DFS **(B)**. According to the Multivariate Cox regression analysis, CD73 could serve as an independent predictor of adverse clinical outcomes **(C)**. The p-values were determined *via* log-rank test.

We found a strong correlation between high expression of CD73 with worse overall survival (OS) and disease-free survival (DFS) in both two sets (P <0.05; **Figure 2A**). However, no correlation between PD-L1 expression and OS or DFS was found in the discovery set, and the significance was evaluated in the independent validation set (P >0.05; **Figure 2B**). Both univariate and multivariate analyses were performed, and the results were detailed in **Table 2** and **Figure 2C**. Univariate analysis revealed that lymph nodes metastasis, clinical stage and CD73 expression were associated with OS and DFS in both cohorts. Multivariate analysis identified CD73 as an independent predictor of both OS and DFS in melanoma patient cohorts.

**Table 2:** Univariate and multivariate analysis of prognostic factors associated with OS and DFS.

Variable	Overall survival						Disease-free survival					
	Discovery set (n=90)			Validation set (n=104)			Discovery set (n=90)			Validation set (n=104)		
	Univariate *p*-value	Multivariate *p*-value	Multivariate HR (95%CI)	Univariate *p*-value	Multivariate *p*-value	Multivariate HR (95%CI)	Univariate *p*-value	Multivariate *p*-value	Multivariate HR (95%CI)	Univariate *p*-value	Multivariate *p*-value	Multivariate HR (95%CI)
Age, year	0.707	NA	NA	0.242	NA	NA	0.971	NA	NA	0.302	NA	NA
(≥60 vs. <60)												
Gender	0.646	NA	NA	0.862	NA	NA	0.746	NA	NA	0.996	NA	NA
(Male vs. Female)												
Anatomic site	0.129	NA	NA	0.519	NA	NA	0.45	NA	NA	0.889	NA	NA
(Acra vs. Trunk vs. Other)												
Histologic type	0.194	NA	NA	0.815	NA	NA	0.86	NA	NA	0.953	NA	NA
(Superfical spreading vs. Nodular vs. Acral vs. Lentigo maligna)												
Breslow depth (mm)	0.151	NA	NA	0.039	0.148	0.497 (0.193-1.281)	0.056	NA	NA	0.034	0.125	0.477 (0.185-1.228)
(>2 vs. ≤2)												
Clark level	0.008	0.294	1.427 (0.735-2.769)	0.405	NA	NA	0.009	0.199	1.536 (0.798-2.957)	0.337	NA	NA
(IV–V vs. I–III)												
Ulceration	0.823	NA	NA	0.707	NA	NA	0.94	NA	NA	0.646	NA	NA
(Present vs. Absent)												
Lymph nodes metastasis	0.001	0.384	0.395 (0.049-3.191)	0.018	0.255	1.581 (0.718-3.479)	0.002	0.938	1.086 (0.135-8.755)	0.031	0.506	1.303 (0.597-2.847)
(Yes vs. No)												
Distant metastasis	<0.001	0.224	0.256 (0.029-2.295)	0.384	NA	NA	0.004	0.64	0.597 (0.069-5.195)	0.249	NA	NA
(Yes vs. No)												
Clinical stage	<0.001	0.011	2.538 (2.070-24.385)	0.004	0.044	1.844 (1.166-3.524)	<0.001	0.22	4.238 (0.423-42.497)	0.004	0.048	1.932 (1.005-3.717)
(III–IV vs. I–II)												
CD73 expression	0.008	0.018	1.548 (1.082-2.941)	0.007	0.024	1.914 (1.089-3.364)	0.002	0.036	1.773 (1.129-3.411)	0.017	0.029	1.618 (1.299-2.819)
(High vs. Low)												
PD-L1 expression	0.388	NA	NA	0.726	NA	NA	0.137	NA	NA	0.955	NA	NA
(High vs. Low)												

HR, hazard ratio; CI, confidence interval; NA, not available.

p < 0.05 was considered significant.

To sum up, according to both discovery and validation sets, high CD73 expression was identified as an independent prognostic factor for both OS and DFS, while PD-L1 failed to stratify OS and DFS in both sets.

### Functional annotation of CD73

**Figure 3:**
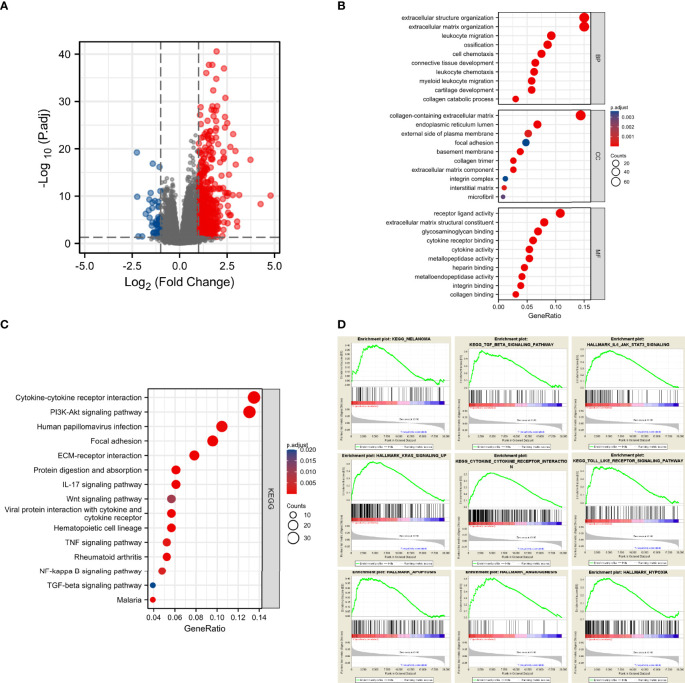
CD73 expression was markedly associated with tumorigenesis and tumor immunity. Significantly altered genes in CD73^high^ tumor shown on volcano plot **(A)**; Bar plots displayed the top 10 BP, MF and CC terms substantially correlated with CD73, and most of them were tumor-related and immune-related **(B)**; The bubble plot showed the top 15 KEGG terms substantially correlated with CD73, and they were also tumor-related and immune-related **(C)**; The results of GSEA revealed that most of the CD73-related pathways were related to tumorigenesis and activation of immune response **(D)**.

Since we found that CD73 was associated with melanoma progression and poor prognosis, we next sought to explore its potential biological function. We did observe 548 genes that were differentially expressed between CD73^high^ and CD73^low^ cohorts in TCGA (**Figure 3A**). Then, we performed enrichment analysis of these genes, and 574 Biological Process (BP) terms, 21 Cellular Component (CC) terms, 53 Molecular Function (MF) terms and 21 KEGG terms were enriched (p<0.05). Several biological functions and signaling pathways were found to be immune-related (**Figures 3B, C**)

To further evaluate the impact of CD73 on signaling pathways, we performed GSEA using RNA-seq data from the TCGA melanoma cohort. A total of 60 positively correlated KEGG and HALLMARK pathways were obtained (FDR<0.25, p<0.05, |NES| >1), including melanoma, TGF-β signaling pathway, IL6-JAK-STAT3 signaling pathway, KRAS signaling up, cytokine-cytokine receptor interaction, Toll-like receptor signaling pathway, apoptosis, angiogenesis and hypoxia (**Figure 3D**). Besides, we explored the impact of CD73 on the enzymatic activity of CD73-CD39 axis and ADO production (**Supplementary Figure 2**). Most of these pathways were tumor and immune-related too, strongly suggesting that CD73 was involved in the TME of melanoma.

### Patterns of tumor-infiltrating immune cells related to CD73 expression

**Figure 4:**
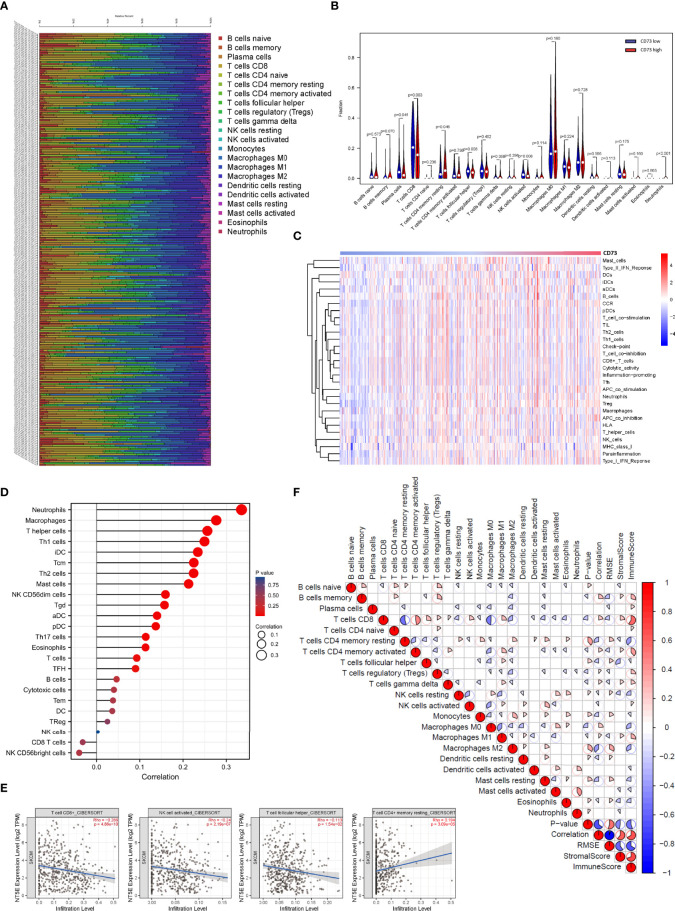
Correlations between CD73 expression and immune infiltration levels in the TCGA cohort. The immune infiltration patterns in 471 tumor tissues arranged by CD73 expression **(A)**. Differential analysis of immune cells between CD73^high^ and CD73^low^ groups in TCGA **(B)**. Detection of the correlation between the CD73 expression level and a series of immune cells using ssGSEA analysis **(C, D)**. CD73 expression was significantly inversely correlated with infiltration of CD8^+^ T cells, activated NK cells and follicular helper T cells, while considerably positively correlated with infiltration of resting memory CD4^+^ T cells **(E)**. Heatmap showed the correlation between representative immune cells infiltration **(F)**.

Here, we investigated the correlation between CD73 expression and immune cell infiltration in melanoma. First, TCGA tumor samples were qualified using the CIBERSORT algorithm. The landscape of immune infiltrations was summarized in **Figure 4A**. To better understand the effect of CD73 on tumor-infiltrating immune cells, we downloaded the RNA-seq profiles of 471 melanoma tumor samples from TCGA database. Then the expression of CD73 of all samples were arranged from low to high, and the median expression of CD73 among the 471 samples was calculated. Then samples with higher expression of CD73 than the median was classified into the high expression group, and those with lower count than the median was defined as the low expression group. Compared with CD73^low^ group, CD73^high^ group was infiltrated with lower proportions of CD8^+^ T cells, activated NK cells and follicular helper T cells, whereas the proportion of resting memory CD4^+^ T cells was relatively higher (p < 0.05 for all, **Figure 4B**). Then, we utilized ssGSEA on RNA-seq data from the TCGA melanoma cohort to estimate the abundance of each immune cell. To fully characterize the effects of CD73 expression on immune cells, we evaluated the abundance of immune cells in patients with high and low CD73 expression. It was found that cytotoxic immune cells were significantly decreased in patients with high CD73 expression, suggesting the function of CD73 in the suppression of immunity (**Figures 4C, D**).

To confirm whether CD73 expression had an impact on the TME, correlation coefficients of CD73 expression and TME infiltrations were calculated using TIMER 2.0. CD73 expression was found to be negatively correlated to immune-active cells including CD8^+^ T cells (Spearman’s r=-0.286, p <0.001), activated NK cells (Spearman’s r=-0.24, p <0.001) and follicular helper T cell (Spearman’s r=-0.113, p =0.0154), while positively correlated to resting memory CD4^+^ T cells (Spearman’s r=0.194, p <0.001) (**Figures 4E, F**). The results above suggested that CD73 expression may have a deep impact on the infiltration of immune cells in melanoma.

### Intratumoral CD73^+^ cells abundance were associated with dysfunctional CD8^+^T cells infiltration in melanoma

**Figure 5:**
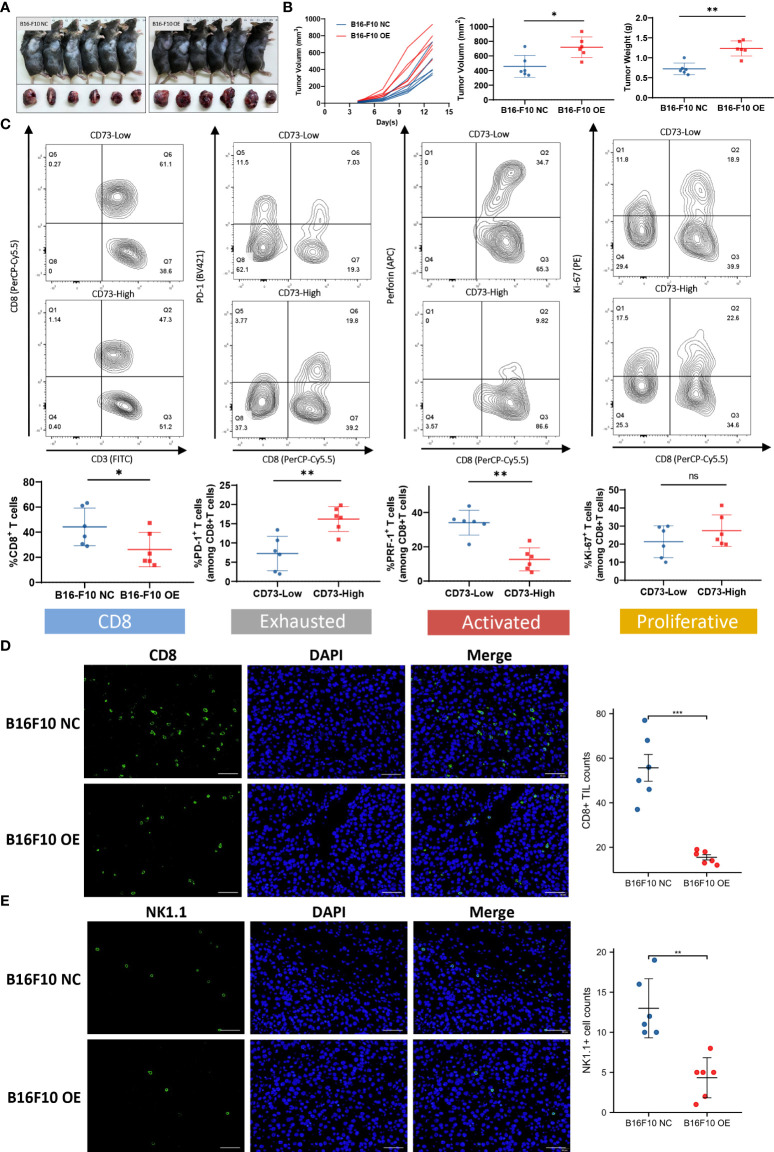
CD73 promoted melanoma growth and was associated with CD8^+^ T cell infiltration and exhaustion. Tumor xenografts of C57BL/6 mice were established by using CD73-NC and CD73-OE B16-F10 cells **(A, B)**. CD8^+^T cell infiltration level in CD73^high^ and CD73^low^ subgroup. Expression of immune exhausted markers, activated markers and proliferative markers for total CD8^+^ T cells in CD73^high^ and CD73^low^ subgroup **(C)**. CD8^+^ T cells and NK1.1^+^ cells infiltrated in CD73-NC and CD73-OE tumors **(D, E)**. *p<0.05; **p<0.01; ***p<0.001; ns, no significance.

To investigate the effect of CD73 on tumor growth *in vivo*, we established tumor xenografts using CD73-NC and CD73-OE B16-F10 cells on C57BL/6 mice (**Figure 5A**). The growth curve showed that overexpression of CD73 in melanoma cells could promote tumor growth (**Figure 5B**). The immune function of CD8^+^ T cells was subsequently detected. The proportion of CD8^+^ T cells was lower in CD73^high^ group (p<0.05). Moreover, these CD8^+^ T cells possessed increased exhausted markers (PD-1) (p<0.01) and decreased activated molecules (PRF-1) (p<0.01). While the proliferation ability (Ki-67) of CD8^+^ T cells showed no significant difference between CD73 ^high/low^ groups (**Figure 5C**). The infiltration of CD8^+^ T cells and NK1.1^+^ cells were evaluated by IF (**Figures 5D, E**) and they were found much lower in CD73-OE group. In conclusion, CD73 promoted tumor progression and inhibited CD8^+^ T cell infiltration and activation in melanoma.

### Tumor-promoting and immunosuppressive function of CD73 could be inhibited by APCP

**Figure 6:**
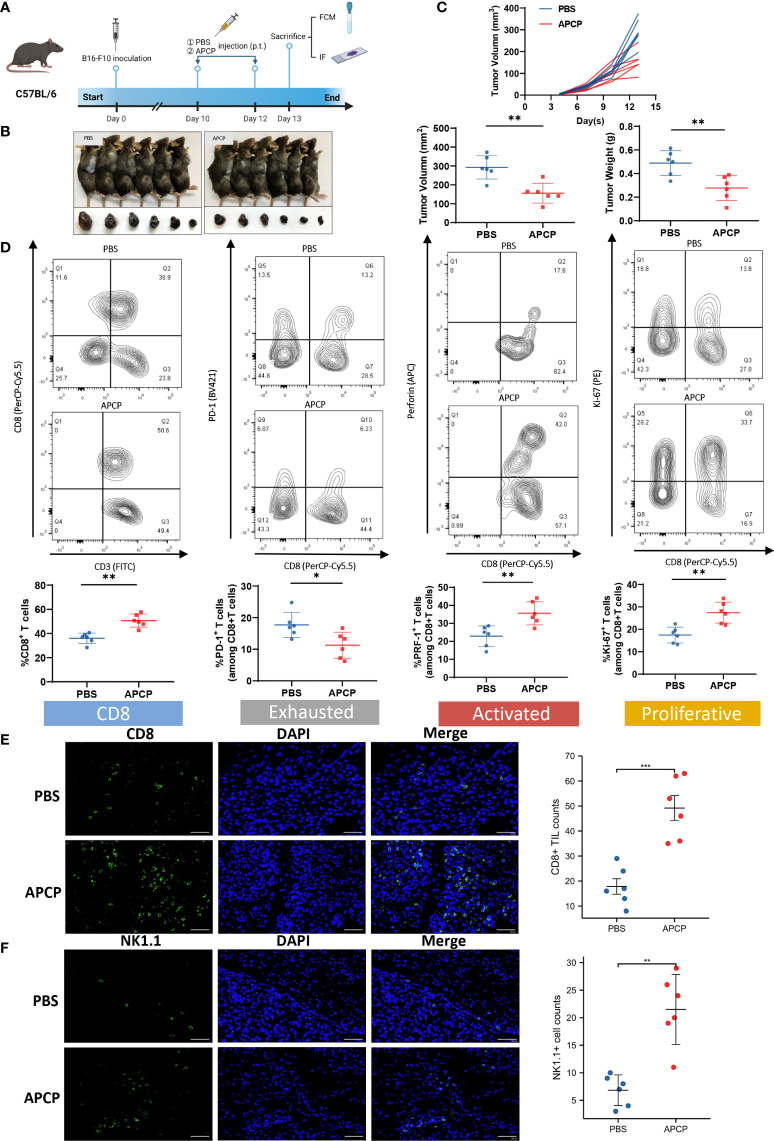
The function of CD73 inhibitor APCP on tumor growth and CD8^+^ T cell infiltration. Tumor xenografts of C57BL/6 mice by using B16-F10 cells were treated with PBS or CD73 inhibitor APCP **(A–C)**. CD8^+^ T cell infiltration level in PBS/APCP subgroup. Expression of immune exhausted markers, activated markers and proliferative markers for total CD8^+^T cells in PBS/APCP subgroup **(D)**. CD8^+^ T cells and NK1.1^+^ cells infiltrated in tumors treated with PBS and APCP **(E, F)**. *p<0.05; **p<0.01; ***p<0.001.

To further investigate the impact of CD73 on TME, we established tumor xenografts using B16-F10 cells, and respectively treated them with PBS or APCP, a small molecule inhibitor targeting CD73 (**Figures 6A–C**). Contrary to the previous results, the proportion of CD8^+^ T cell was elevated in the APCP group (p<0.01), with lower PD-1 expression (p<0.05) and higher PRF-1 and Ki-67 expression (p<0.01) (**Figure 6D**). The infiltration of CD8^+^ T cells and NK1.1^+^ cells were increased significantly following APCP treatment (**Figures 6E, F**). In conclusion, the tumor-promoting and immune-suppressive function of CD73 in melanoma could be blocked by CD73 inhibitor APCP.

### Tumor-infiltrating immune cells and their associations with CD73 and PD-L1 expression

**Figure 7:**
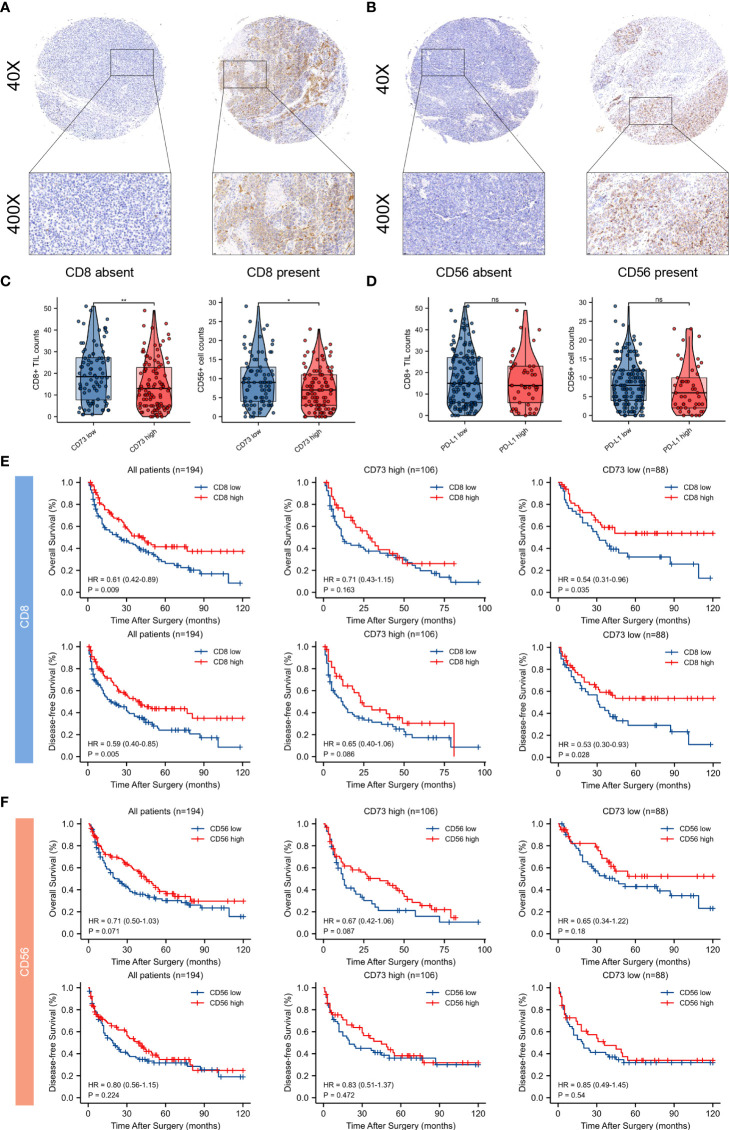
Tumor infiltrating CD8^+^ T cells and CD56^+^ NK cells and their correlations between CD73 and PD-L1 expression, and prognostic value of intratumoral immune cells in CD73^high^ and CD73^low^ subgroups. Typical micrographs of positive CD8 **(A)**, CD56 **(B)** staining and the corresponding intra-tumor negative controls. Original magnification × 40 for full views and × 400 for detailed views. Scatter plots depicted the correlation between CD8^+^ T cells and CD56^+^ NK cells and CD73 expression. High CD73 expression. was significantly correlated with lower intra-tumor counts of CD8^+^ T cells and CD56^+^ NK cells **(C)**. PD-L1 expression had no significant correlation with counts of CD8^+^ T cells and CD56^+^ NK cells **(D)**. Kaplan-Meier curves of OS and DFS in CD73^high^ and CD73^low^ subgroups according to CD8^+^ and CD56^+^ cells infiltration **(E, F)**. *p<0.05; **p<0.01; ns, no significance.

Typical micrographs of CD8^+^ T cells and CD56^+^ NK cells, which represented cytotoxic immune cells, were presented in **Figures 7A, B**. High CD73 expression was correlated with lower intratumoral CD8^+^ T cells and CD56^+^ NK cell counts (P < 0.01 and P < 0.05, respectively; **Figure 7C**). In contrast to an immunosuppressive TME found in melanoma with elevated CD73, no significant correlation was observed between PD-L1 expression and CD8^+^ T cells and CD56^+^ NK cells counts in melanoma (**Figure 7D**).

In addition, survival analysis was performed to demonstrate the clinical significance of these tumor-infiltrating immune cells in CD73^high^ and CD73^low^ melanoma. Remarkably, CD8^+^ T cell infiltration predicted distinct survival outcomes in CD73^high^ and CD73^low^ groups, indicating that the survival benefits from high infiltration of CD8^+^ T cells might be diminished by CD73. However, Kaplan-Meier curves showed no significant differences between CD56^+^ NK cells within two groups (**Figures 7E, F**).

## Discussion

TME is a complex ecosystem involving tumor cells, immune cells, extracellular matrix and other factors (35). Considerable efforts have been made in recent years to assess the prognostic value of various immune cells within the TME. Previous studies have demonstrated that tumor-infiltrating immune cells played distinct roles in tumor progression in multiple cancer types (36–39). The TME landscape and the interactions between tumor cells and TME during tumorigenesis and its progression could imply the response to immunotherapy (40). Thus, it is essential to elucidate the characteristics of TME in specific subgroups of melanoma patients for improving prognosis prediction.

CD73, also known as ecto-5’-nucleotidase (*NT5E*), functions as a novel immune checkpoint by producing eADO, which subsequently inhibits immune activation by interacting with the downstream receptor of the adenosine pathway (21). In this study, we observed that CD73 was more ubiquitously expressed than PD-L1 in melanoma and CD73 overexpression was prevalent in PD-L1 negative melanoma. Studies of Monteiro et al. observed that CD73 expression in 54% of metastatic melanoma, and CD73 expression in tumor cells significantly correlated with decreased OS (41). Young et al. reported no significant association was identified between positive CD73 protein expression and survival. They suggested that CD73 expression was not an independent prognostic factor in melanoma, while it was positively associated with the advanced stage of melanoma (42). Reinhardt et al. reported that CD73 showed variable expression in human melanoma, and CD73 failed to stratify OS and PFS (43). In our study, the high expression rate of CD73 were 55.6% and 53.8% in two melanoma cohorts, respectively. Additionally, we found that patients with high CD73 expression had significantly poorer OS and DFS. Intriguingly, CD73 displayed heterogeneity in its expression pattern in melanoma, and the prognostic significances of CD73 were inconsistent among different studies. The discrepancy between these and our results may be caused by the following reasons. Firstly, subtypes of melanoma are strikingly different between the East and West. Cutaneous melanoma dominates in Caucasian while acral and mucosal melanomas were more common in Asian populations (44). In Monteiro’s study, cutaneous melanoma accounted for 79% in their cohorts, while acral melanoma consisted of more than 55% of all cases in our study, which mainly attributed to the different CD73 expression rate across studies. Moreover, the sample size of our research was larger than that of the others, which improved the credibility of the results. Lastly, elevated CD73 expression in the tumor tissue has been reported to be associated with advanced clinical stage and poor prognosis in several cancer types (23, 45, 46), which underscores the crucial role of CD73 in tumor progression. Therefore, it is reasonable to speculate that CD73 is correlated with poor clinical outcomes in melanoma.

BRAF mutational status is usually an important factor for melanoma. Although BRAF mutation occurs in about 50% of the melanoma cases around the world (16, 47), the mutation rate of BRAF is much lower among patients in China, which is about 23-29% (17, 48–50). Due to the detection of BRAF mutation were unavailable among patients in our cohorts, we additionally explored the correlation between CD73 expression and the BRAF mutational status utilizing data from public database (GSE158403 and GSE190113). Notably, the cohorts of GSE190113 consisted of patients with acral melanoma, which was in accordance with the main subtype for Chinese population. However, we failed to find significant correlation between CD73 expression and BRAF mutation in both datasets. It is worthy of further exploration on the impact of BRAF mutation together with CD73 on the prognosis of Chinese patients in the future study. Besides, Young et al. combined adenosine A2A receptor (A2AR) antagonist with BRAF and MEK inhibition in melanoma cell lines and murine melanoma BRAF-mutated models (51). They found that tumor growth and metastasis were reduced in inducible and experimental BRAF-mutant melanoma treated with the combination therapy targeting BRAF and A2AR. This study has demonstrated that targeting adenosine pathway combined with BRAF inhibition enhanced immune responses to metastatic melanoma. This is an inspiration for us to investigate the combination of CD73 inhibition, since CD73 is the rate-limiting enzyme of adenosine production.

CD73 has been reported to shape an inhibitory tumor microenvironment and reduce anti-tumor immunity in a variety of tumors (52–54). For melanoma, acral and mucosal melanomas are more common subtypes in Asian population. Since the clinical efficacy of PD-1 blockade has not been satisfying in acral and mucosal melanomas, our study explored the role of CD73 in the TME in Chinese melanoma patients. Our study also revealed that there was significant difference in the patterns of immune cell infiltration related to CD73 and PD-L1. CD73 overexpression was significantly correlated with sparser CD8^+^ T cells and CD56^+^ NK cells. In addition to reducing CD8^+^ T cell infiltration, CD73 might also cause T cell exhaustion. Majority of the CD8^+^ T cells in CD73^high^ tumors exhibited a dysfunctional phenotype with decreased CD8^+^ T cells infiltration and perforin-1 level, yet elevated PD-1 expression. However, no significant correlation was observed between PD-L1 expression and these two immune cells. The infiltration of CD8^+^ T cells is known as a predicting factor for favorable survival, which was also verified in our study. As a result of the exposure to suppressive gradients in TME, CD8^+^ T cells gradually formulated their exhaustion states (55). Consequently, CD8^+^ T cell exhaustion might play an essential role in immune evasion in CD73 enriched melanoma. In addition, CD73 may serve as a target to be combined with other immunotherapies, which has been reported in pre-clinical models in other cancer types (56, 57), supporting that combination therapy of CD73 inhibition and current melanoma immunotherapies is worth exploring. Collectively speaking, CD73 expression was associated with an inhibitory TME characterized by attenuated cytotoxic T lymphocytes (CTLs) and NK cells, indicating the potential role of CD73 as an immunotherapeutic target.

Melanoma is a lethal malignancy with limited therapeutic options. With the initial approval of pembrolizumab and nivolumab in 2014–2015, the treatment of advanced melanoma has been revolutionized by the clinical development of PD-1 blockade. Trials of PD-1 blockade focusing on cutaneous melanoma have demonstrated long-term objective response rates (ORR) of approximately 45%, with more than 40% patients surviving for over 5 years (19, 58). However, the response rate was estimated to be much lower (23%–32%) for patients with acral and mucosal melanoma (59),which were more common subtypes in China (48).

Due to molecular, genomic and immunologic differences, efficacy of PD-1 blockade tended to be lower within Chinese population compared to Western population (60). Insufficient T cell infiltration may partly be responsible for the poor response to immunotherapy of some patients (61). Our study hypothesized that CD73 could decrease the infiltration of cytotoxic immune cells, formulate its exhausted state and shape an inhibitory TME, thus inducing resistance to immunotherapy.

CD73 is reported to promote cellular adhesion, angiogenesis and the migration of malignant cells. Most importantly, CD73 is the rate-limiting enzyme converting ATP into adenosine, while the accumulation of adenosine in the TME has been proved to promote tumor progression by mediating immunosuppression and dampening the anti-tumor function of immune cells (62). The immunosuppressive effect of adenosine on the TME is also considered as a crucial role contributing to the resistance to anti-PD-1/PD-L1 therapy (63, 64). The restriction of anti-PD-1/PD-L1 efficacy mediated by adenosine has suggested the strategy of combining inhibition of adenosine metabolism and anti-PD-1/PD-L1 therapy, which requires to be further investigated in our future study.

Honestly, there are some flaws in our current research and follow-up research are required. For example, the discovery and validation sets were both derived from one single center, therefore the expression pattern of CD73 and PD-L1 of melanoma patients in other cohorts are subject to further studies. In addition, the xenograft tumor models could not well duplicate the microenvironment of the orthotopic tumor. TME was a sophisticated system and its modification in melanoma was multilayered and multicell participated, which deserved further exploration. Our study was retrospective and our conclusions required to be confirmed by further validation in larger-scaled and multi-centered clinical cases.

**Figure 8:**
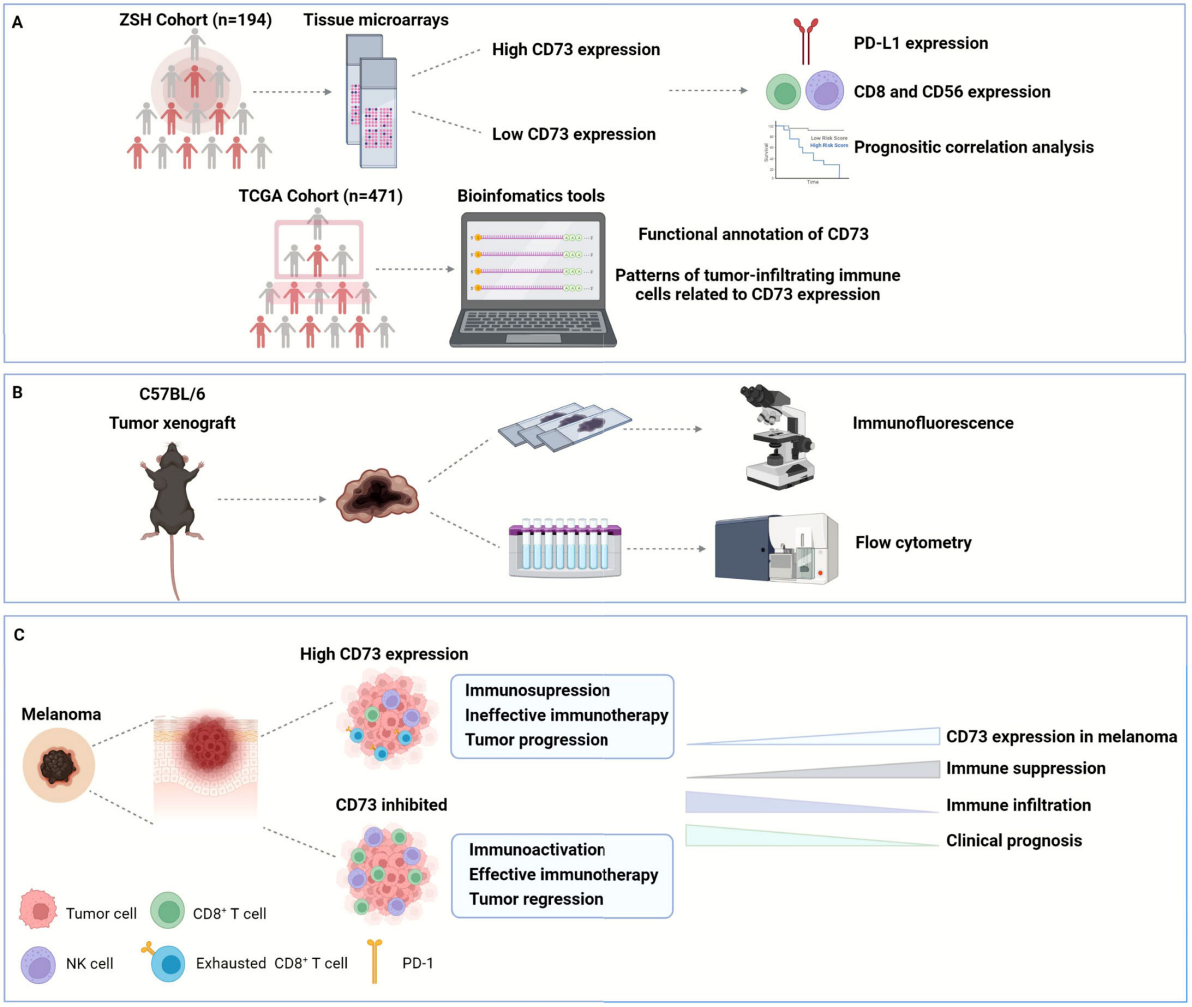
Graphical abstract. In this study, we enrolled two cohorts of 194 melanoma patients from Zhongshan Hospital to explore the expression of CD73, PD-L1, CD8 and CD56 in their tumor tissues, and confirmed the poor OS and DFS in CD73^high^ patient subgroup. We also investigated the functional enrichment and patterns of infiltrating immune cells related to CD73 by analyzing RNA-seq of 471 melanoma patients from TCGA **(A)**. Furthermore, we established tumor xenograft of melanoma to explore the role of CD73 in tumor progression and its impact on TME **(B)**. CD73^high^ tumors showed an inhibitory TME with fewer CD8^+^ T cells infiltration displaying a dysfunctional phenotype, which could be reactivated when treated with CD73 inhibitor **(C)**. OS, overall survival; DFS, disease-free survival; TCGA, the Cancer Genome Atlas; TME, tumor microenvironment.

In this study, we identified CD73 expression as an independent prognostic factor, and high CD73 expression predicted poor patient outcome in melanoma (**Figures 8A–C**). We compared the clinical outcomes between CD73^high/low^ subgroups to investigate the prognostic value of CD73 in melanoma. In both of the discovery and validation cohorts, CD73^high^ subgroups had significantly poorer OS and DFS. And we also looked into the expression pattern of CD73 and PD-L1 in melanoma. Compared to PD-L1, CD73 was more commonly expressed in melanoma cases and was also frequent in PD-L1 negative cases, suggesting that CD73 possessed more significant prognostic value. Furthermore, we performed univariate and multivariate Cox regression analysis to determine the potential independent prognostic factors for the survival outcomes of patients with melanoma, and we identified CD73 as an independent prognostic factor. We explicated that overexpression of CD73 contributed to an inhibitory TME by suppressing CD8^+^ T cells and CD56^+^ NK cells and inducing its exhaustion states which could be blocked by CD73 inhibitor. Since we mainly enrolled acral melanoma in our cohorts, which represents the most common subtype in Asians, it is reasonable to believe that CD73 may represent an ideal immunotherapy target next to PD-L1 in melanoma.

## Data availability statement

The names of the repositories and accession numbers can be found in the article/**Supplementary Material**.

## Ethics statement

This study was approved by the Ethics Committee of the Zhongshan Hospital Biomedical Research Department, and written informed consent was obtained from all participants. The animal study was reviewed and approved by the the Animal Experimentation Ethics Committee of Zhongshan Hospital, Fudan university. Written informed consent was obtained from the individual(s), and minor(s)’ legal guardian/next of kin, for the publication of any potentially identifiable images or data included in this article.

## Author contributions

JG and CW designed and conceived this study. ZG, LW, ZS, and MR performed experiments, analyzed data and wrote the manuscript. YY, JL, KS, and YL conducted the statistical analyses. YD and YWY analyzed the data and collected tumor samples. JG, CW, and YZ supervised the study. All authors contributed to the article and approved the submitted version.

## Funding

This study was funded by the National Natural Science Foundation of China (grant no. 81972559), the Shanghai Municipal Natural Science Foundation (grant no. 22YF1407400), and the Shanghai Shenkang Hospital Development Center Project (project no. SHDC2020CR2067B).

## Conflict of interest

The authors declare that the research was conducted in the absence of any commercial or financial relationships that could be construed as a potential conflict of interest.

## Publisher’s note

All claims expressed in this article are solely those of the authors and do not necessarily represent those of their affiliated organizations, or those of the publisher, the editors and the reviewers. Any product that may be evaluated in this article, or claim that may be made by its manufacturer, is not guaranteed or endorsed by the publisher.
