# Supplementary material for: Intratumoral CD73: An immune checkpoint shaping an inhibitory tumor microenvironment and implicating poor prognosis in Chinese melanoma cohorts
Source: Front Immunol. 2022 Sep 5;13:954039. doi: 10.3389/fimmu.2022.954039 (PMC9483101; doi:10.3389/fimmu.2022.954039)
Supplement: Supplementary file 1 [file DataSheet_1.pdf]

Supplementary Materials

Supplementary Table 1. Antibodies applied for immunohistochemistry, immunofluorescence and flow cytometry.

No.	Antibody Name	Description	Manufacturer	Dilution/Dose	Application
1	Anti-CD73 antibody	Rabbit monoclonal	CST	1:200	IHC
2	Anti-PD-L1 antibody	Rabbit monoclonal	Abcam	1:500	IHC
3	Anti-CD8 antibody	Rabbit monoclonal	Abcam	1:100	IHC
4	Anti-NCAM1 (CD56) antibody	Rabbit monoclonal	CST	1:100	IHC
5	Anti-CD8 antibody	Rabbit monoclonal	CST	1:100	IF
6	Anti-CD161c antibody	Rabbit monoclonal	CST	1:200	IF
7	FITC Anti-CD3 antibody	Mouse monoclonal	BD Biosciences	1μl	FCM
8	PerCP-Cy5.5 Anti-CD8 antibody	Mouse monoclonal	BD Biosciences	1μl	FCM
9	BV421 Anti-CD279 (PD-1) antibody	Mouse monoclonal	BD Biosciences	1μl	FCM
10	APC Anti-Perforin antibody	Mouse monoclonal	Biolegend	1μl	FCM
11	PE Anti-Ki-67 antibody	Mouse monoclonal	Biolegend	1μl	FCM
12	Anti-CD73 antibody	Rabbit monoclonal	CST	1:1000	WB
13	HRP-conjugated GAPDH antibody	Mouse monoclonal	Proteintech	1:10000	WB
14	Anti-CD39 antibody	Rabbit monoclonal	Abcam	1:1000	WB
15	Anti-Adenosine Receptor A2a antibody	Mouse monoclonal	Abcam	1:500	WB

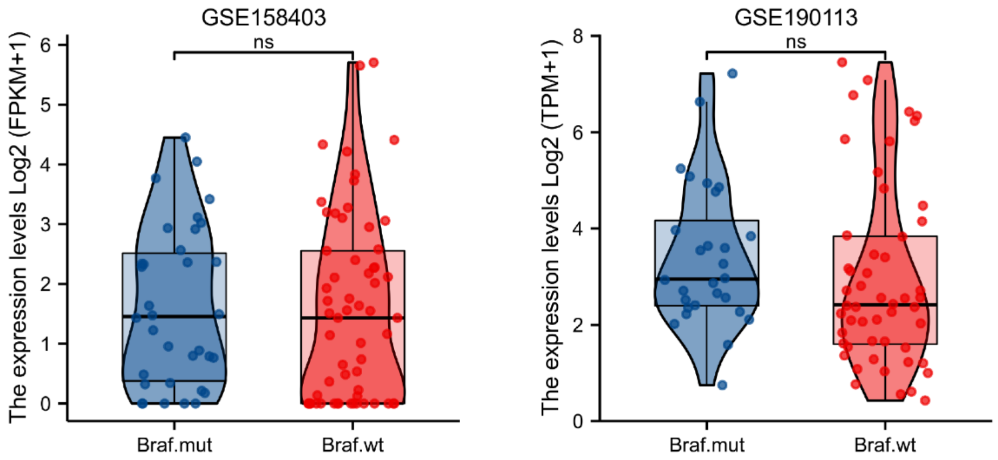

Supplementary Figure 1. CD73 expression levels of BRAF mutated and wild-type patients in GSE158403 and GSE190113.

There is no significant correlation between CD73 expression and BRAF mutation in GSE158403 and GSE190113.

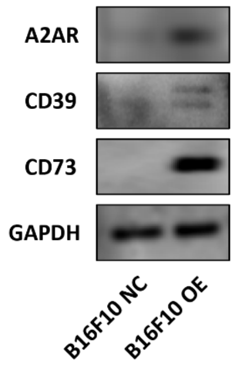

Supplementary Figure 2. Impact of CD73 on the enzymatic activity of CD39-CD73-A2AR axis by western blot.

CD39 and A2AR are upregulated in CD73-overexpressing group in B16F10 compared with the control group.
